# RE-Based Inorganic-Crystal Nanofibers Produced by Electrospinning for Photonic Applications

**DOI:** 10.3390/ma14102679

**Published:** 2021-05-20

**Authors:** Alessandra Toncelli

**Affiliations:** 1 Dipartimento di Fisica “E. Fermi”, Università di Pisa, Largo B, Pontecorvo 3, 56127 Pisa, Italy; alessandra.toncelli@unipi.it; 2 Istituto Nazionale di Fisica Nucleare, Sezione di Pisa, Largo B, Pontecorvo 3, 56127 Pisa, Italy; 3 Istituto Nanoscienze—CNR, Piazza S. Silvestro 12, 56127 Pisa, Italy

**Keywords:** electrospinning, rare earth, nanofibers, upconversion, fluoride crystals, oxide crystals, photonic applications

## Abstract

Electrospinning is an effective and inexpensive technique to grow polymer materials in nanofiber shape with exceptionally high surface-area-to-volume ratio. Although it has been known for about a century, it has gained much interest in the new millennium thanks to its low cost and versatility, which has permitted to obtain a large variety of multifunctional compositions with a rich collection of new possible applications. Rare-earth doped materials possess many remarkable features that have been exploited, for example, for diode pumped bulk solid-state lasers in the visible and near infrared regions, or for biomedical applications when grown in nanometric form. In the last few decades, electrospinning preparation of rare-earth-doped crystal nanofibers has been developed and many different materials have been successfully grown. Crystal host, crystal quality and nanosized shape can deeply influence the optical properties of embedded rare earth ions; therefore, a large number of papers has recently been devoted to the growth and characterization of rare earth doped nanofibers with the electrospinning technique and an up-to-date review of this rapidly developing topic is missing; This review paper is devoted to the presentation of the main results obtained in this field up to now with particular insight into the optical characterization of the various materials grown with this technique.

## 1. Introduction

**Figure 1:**
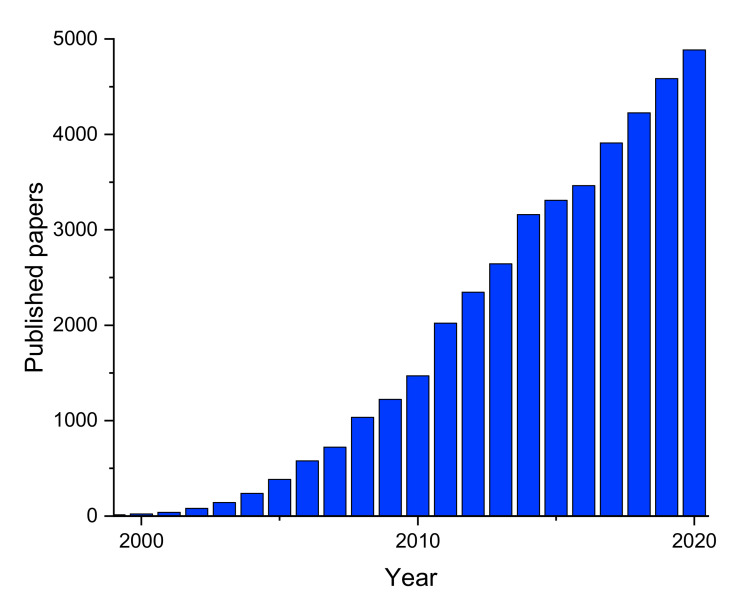
Trend of the annual number of scientific publications in the new millennium as obtained from the Scopus database using the search term “electrospinning” in April 2021.

Since the discovery of carbon nanotubes, the interest in developing new techniques to grow nanosized materials has continuously been growing because the peculiar physico-chemical properties of one-dimensional materials lead to many promising applications in different fields such as optics, electronics, catalysis, gas capture, biology, filtration, etc. Several methods have been developed for the growth of nanosized materials [1], like hydrothermal growth, thermal evaporation, arc discharge, chemical vapor deposition and electrospinning. Every method has its own advantages and disadvantages, but among all, electrospinning excels in terms of cost, time, ease of operation and permits to obtain 1-dimensional (1-D) structures with unique features. This technique has been developed at the beginning of the XX century. Despite its long story, it has received renewed attention in the new millennium, as demonstrated from the annual trend of scientific publications obtained from the Scopus database in March 2021 by searching the keyword “electrospinning” and shown in Figure 1. In these last two decades, this technique has experienced a strong technical development which has permitted to obtain many different polymer nanofibers with diameters in the micrometer to nanometer range with length-to-radius ratio up to 10^10^.

Electrospun fibers possess a series of remarkable characteristics and most of them are connected to their ultra-high surface-to-volume ratio; moreover, they can have superior mechanical properties as for stiffness, tensile strength and flexibility in their composition that can be based on both organic and inorganic polymers [2,3]. All these features, together with the additional benefit of a variety of possible shapes and the possibility of surface functionalization, make them interesting candidates for many applications [4,5,6]. For example, they have already been proposed for electronic applications such as micro/nano electronic devices, electromagnetic shields, nano solar cells, LCD devices, capacitors and fuel cells, for mechanical applications as ultra-lightweight spacecraft materials, for chemical applications as functional catalysts, for security as nano sensors, protective clothing and filtering, bio-medical applications as tissue engineering scaffolding, drug delivery carriers, hemostatic devices and many more [7].

In its simplest implementation, electrospinning is based on the continuous and controlled flow of a polymer solution through a nozzle and the acceleration of the solution by a high voltage applied between the nozzle and the collector unit. A jet is readily formed and collected on a plate at ground potential. The great development of the technique in the last few decades has permitted the optimization of the growth parameters like the voltage, the polymer flow, the needle dimension, the distance between the nozzle and the collector, etc. to obtain materials of very high quality. At the end of the growth process, nanosized fibers with uniform diameter can be collected in random directions with a simple collector plate or can be aligned with the help, for example, of a rotating drum. Other interesting developments include the use of composite needle systems to obtain structured fibers with multifunctional capabilities. The main limitation of the method is the need to start from a solution with the right viscous properties to be extruded and to avoid instabilities of the extruded jet before collecting. This limits the choice of the material and usually leads to the formation of amorphous fibers, but a careful optimization of the materials and/or aftergrowth annealing, can permit to obtain crystal fibers or polymer fibers with nanocrystals embedded inside.

Rare earth (RE) ions have been studied both as free ions and as dopants in inorganic crystals thanks to their extraordinary spectroscopic features. In fact, they have metastable energy levels that show bright emissions from UV to the mid infrared regions that have been exploited for a variety of photonic applications, including the development of diode-pumped-solid-state lasers (DPSSL) in the visible and infrared regions [8,9,10]. Thanks to the long lifetime of their metastable levels, RE ions also give rise to anti-Stokes emissions based on an energy transfer process called upconversion which promotes the excitation to higher energy levels with minimal losses. This phenomenon has become very popular in RE-doped nanoparticles, especially fluoride nanoparticles, that can show very bright anti-Stokes emission [11]. When used for bioimaging, these nanoparticles have great advantages over the more common quantum dots, such as fluorescence stability, absence of photobleaching, strong penetration ability, low induced photodamage, weak autofluorescence background, high detection sensitivity and signal-to-noise ratio [12]. For these reasons, the possibility to combine the peculiar chemical-physical features of electrospun fibers with the unique optical characteristics of rare earth ions has the fascinating potential to obtain new types of multifunctional materials [13,14]. Unfortunately, RE ions in polymer matrixes usually exhibit low emission efficiency and the direct growth of crystal fibers through electrospinning is not possible. Instead, a polymeric precursor can be used (usually polyvinylpyrrolidone (PVP)) to obtain a solution with the right viscous properties for the technique and the polymer can be eventually eliminated with a subsequent calcination process which can take place at temperatures lower that those usually required for the solid-state growth of the crystal host. This is the preferred approach when growing oxide crystal nanofibers. The growth of fluoride crystal nanofibers needs an additional fluorination step after the calcination process. This complicates the procedure also because the fluorination process involves the use of dangerous reagents. For this reason, another popular approach is to embed fluoride crystal nanoparticles into polymer fibers.

This review presents the main scientific results in the growth of rare-earth doped nanofibers with the electrospinning technique with an insight into their luminescence characterization in the visible spectral region. The review is organized as follows: first, an introduction to the physics of rare earth ions in crystals is given with insight into the differences between oxide and fluoride crystals. Then, the basics of the electrospinning technique is presented with the description of the strategies used to grow oxide and fluoride nanofibers; finally, the main results of electrospinning growth of rare earth doped crystalline fibers with luminescent properties are presented. These results are organized as a function of the doping ions, with the first section devoted to the ions that show Stokes emission in the UV-VIS region (Eu, Sm, Dy, Nd, etc.) and the following section devoted to upconverting ions (Er, Tm, Ho). The last section is devoted to the presentation of the results obtained by embedding rare earth doped crystal nanoparticles into electrospun polymer fibers.

## 2. Rare Earth Ions in Ionic Crystals

Rare earth ions, also called Lanthanide ions, are located between Lanthanum and Lutecium in the periodic table of the elements and are characterized by an outer electronic configuration 5s^2^ 5p^6^ 4f^n^. In their trivalent state n varies from 1 (Ce^3+^) to 13 (Yb^3+^). This partial filling of the 4f orbital determines the spectroscopic properties of these ions. Rare earth free ions possess a series of energy levels that come from the splitting due to electron-electron interaction and spin-orbit interaction within the 4f shell with energy separations of the order of a few thousand cm^−1^. The number of these energy levels is determined by the number of electrons in the 4f shell and is equal to the binomial coefficient $\left(\begin{array}{c} n\\ 14 \end{array}\right)$. This simple rule tells us that the ions at the extremes of the list have very few energy levels (just one for Ce^3+^ and Yb^3+^) and this number increases towards the middle of the list, but their location can only be predicted with theoretical calculations. Since they belong to the same shell, electric dipole transitions between these levels are strictly parity forbidden. When found in an ionic crystal site as substitutional dopants, they are subject to the crystal field with the symmetry properties of the point site they are in. This crystalline field is partially shielded by the outer 5s and 5d electrons which are spatially larger, but energetically lower than 4f orbitals. This weak crystal field causes a mixing of the 4f orbital with the 5s and 5d orbitals which, in turn, splits the levels, usually called multiplets, in a set of sublevels with energy separations of the order of a few hundred cm^−1^. This weak mixing breaks the parity symmetry and makes electric dipole transitions permitted. Since they are permitted by the weak crystal field, these transitions have low cross sections (of the order of 10^−19^–10^−20^ cm^−1^) and long lifetimes (typically from 10 μs to 10 ms), for this reason they are called forced electric dipole transitions. This gives rare earth ions their peculiar properties, in fact, these long lifetimes permit a large energy storage in the multiplets that gives rise to intense emissions and to peculiar energy transfer processes among the multiplets. The most famous among these processes is called upconversion and happens when two excited ions exchange their energy and, as a result, in the end one of the two ions goes to a higher energy level and the other decays to a lower one. The ion that gains energy can eventually decay to the ground state and emit a photon at a shorter wavelength than that of the pump beam. This anti-Stokes emission, for example, eliminates the background from cellular autofluorescence and this explains the popularity of rare-earth doped crystal nanoparticles for bio-imaging.

Oxide crystals are the preferred hosts for rare earths when the main aim is to obtain Stokes emission in the visible or near infrared region especially if high power densities are involved. This is because oxide crystals are easy to grow and have very good thermomechanical properties, like high thermal conductivity and threshold damage and have already been proposed and used for diode pumped solid state lasers in the visible and near infrared regions, lighting, field emission displays (FED), cathode ray tubes (CRT), plasma display panels (PDP), solar cells and many other applications.

The relatively high phonon energy of oxide crystals (of the order of 1000 cm^−1^) increases the probability of non-radiative emissions that quench low-energy emissions and up-converting processes. Therefore, they are not the best choice when interested in infrared emissions or in bilinear processes. To this aim, fluoride crystals are the most popular host materials for rare earth ions because their wide band gap and the relatively low phonon energy (compared to oxides) minimize non radiative losses inside the material and give rise to very efficient emissions with particularly long lifetimes. This is the case, for example, for the near-infrared emissions of Tm and Ho. In fact, fluoride crystals are the preferred hosts for near- and mid- infrared lasers [10]. At the same time, long fluorescence lifetimes make upconversion processes more probable and maximize the upconverting efficiency of rare earth ions. Therefore, fluoride nanoparticles are usually considered the best 1D upconverting nanomaterial and, among all, NaYF_4_ is considered the best host crystal in this respect [15] and have already been used in biological labelling. Despite the popularity of fluoride materials as bulk crystal hosts and as nanoparticles, the electrospinning growth of fluoride fibers is still in its infancy given the much lower number of published papers with respect of oxide materials, but very interesting results in terms of possible applications have already been presented. The main reason probably lies in the difficulty in growing this type of materials. When grown in bulk crystal form, fluoride materials need very high purity of the starting materials with careful control of the growth atmosphere because even very low levels of impurities strongly affect the emission efficiency of rare earth ions. Electrospinning growth of this type of materials is usually performed through fluorination of oxide electrospun fibers. This implies a further step that involves the use of dangerous chemicals and this is probably the reason why the electrospinning growth of fluoride fibers has received much less attention in terms of number of published papers. Another possible approach is embedding fluoride nanoparticles into polymer fibers obtained through electrospinning. The bottom-up growth of nanoparticles has been optimized to obtain high quality monodisperse nanoparticles [16] that have already been assessed for many different applications [11]. Incorporating these high-quality nanoparticles into polymeric fibers is probably the easiest strategy to obtain highly efficient upconverting nanofibers bypassing the inherent difficulties in the electrospinning growth of fluoride crystal matrixes.

## 3. The Electrospinning Technique

**Figure 2:**
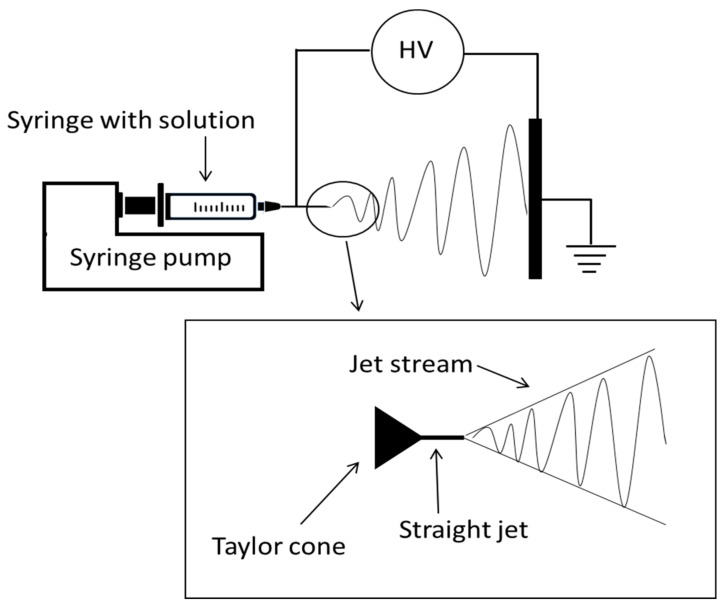
Schematic diagram of the electrospinning setup.

Electrospinning is a simple method to grow materials. The technique is based on the continuous extruding of a polymer material from a needle. The simplest setup is represented in Figure 2. A solution with the right viscoelastic properties is loaded into a syringe and is extruded with a precision pump which permits continuous flows of the solution at very low rates. When the material exits the needle, it experiences the high voltage (typically of the order of 10 kV) that accelerates it towards the collector. This leads to the formation of the so-called Taylor cone. Under appropriate conditions of field gradient, the Taylor cone ends up in a jet stream that is directed towards the collector. During its flight, the jet stream is subjected to different forces (Coulomb force, viscoelastic forces, surface tension forces, gravitational force) that cause instabilities in the jet stream. The jet then, follows a complex path towards the collector and, eventually, nanofibers are randomly deposited on the collector. Thanks to the large surface-to-volume ratio the solvent evaporates from the solution even in the typically short time of flight; therefore, solid fibers can readily be grown. Moreover, the alignment effect of the strong electric field and the high draw-ratio permit to obtain crystalline fibers under proper conditions.

Particular care must be devoted to the homogenization of the solution because this is one of the most important parameters that affects the optical and mechanical properties of the grown fibers. To accomplish this, a polymeric precursor is needed and in most cases PVP is used with just a few exceptions that involve the use of a different polymer material like poly (ethylene oxide) (PEO), polyvinyl alcohol (PVA) or poly lactic acid (PLA). Unfortunately, no systematic study on the influence of the polymeric precursor on the quality of the crystal fibers has been carried out, therefore, the choice of the solution with the right viscoelastic properties is still based on the personal experience of the experimenter. Many other growth parameters must be optimized to control the quality and morphology of the grown materials, for example, the starting solution composition, the flow rate, the voltage and the needle-collector distance. Moreover, other less-direct parameters can have a large influence on the fiber quality, for example the collector temperature and atmosphere humidity [17,18]. When growing nanoparticle/composite fibers aggregation of the nanoparticles must be avoided. A good strategy can be skipping nanoparticle drying steps and using particularly long stirring and ultrasonication times.

**Figure 3:**
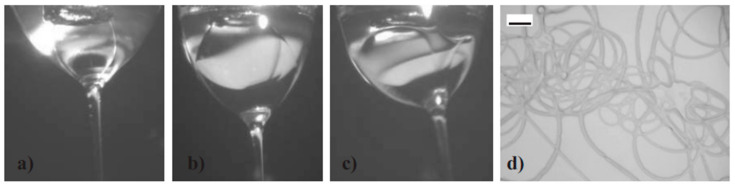
Evolution of PVP–oil Taylor cone geometry. Outer and inner flow rates are: (**a**) 0.4–0.04 mL/h; (**b**) 0.4–0.2 mL/h; (**c**) 0.4–0.5 mL/h. Inner needle OD is 0.5 mm. (**d**) Oil covered microfibers as a result of *Q*out/*Q* in ≈ 1. Scale bar: 20 μm. Reprinted with permission from [28]. Copyright © 2006 WILEY–VCH Verlag GmbH & Co. KGaA, Weinheim.

In the last few decades, the simple technique described above has been developed and engineered to obtain micro- and nanomaterials beyond the simple nanofiber structure like ribbon-shaped nanocables, nanobelts [19], Janus nanofibers [20], Janus nanobelts [21], hollow nanofibers [22], 2D and 3D aligned arrays of nanofibers [23], coaxial nanofibers [24], coaxial nanoribbons [25], nanofibrous membranes [26], etc. Each of them with peculiar properties. Among these, for example, hollow nanofiber morphology permits to double the surface area compared with common solid nanofibers and this can be exploited for surface-related applications such as chemical sensors or photocatalysis. Core-shell nanostructures are particularly interesting for nanoelectronic applications (with an external insulating sheath and a conductive core inside), integrated optics for realizing waveguides and for nanofluidic and biological applications. Hollow-core or core-shell nanofiber growth is usually accomplished using a dual nozzle spinneret with a smaller capillary inside a larger coaxial one [27,28]. As an example, Figure 3 shows the evolution of the Taylor cone geometry during the growth of a PVP–oil nanofiber system with a dual nozzle spinneret as a function of the flow rate.

Another intriguing possibility of this technique is the growth of multifunctional materials. Historically, the technique has been developed for the growth of materials with properties other than optical, but rare earth-doped materials are studied mainly for their luminescent behaviours. The possibility to combine these optical properties with, for example, magnetic properties or drug delivery capabilities is very interesting but difficult to accomplish with high efficiency. For example, the combination of magnetic with luminescent properties often yields to low emission efficiency, because magnetic materials are not the preferred hosts for luminescent centers [19]. Fluoride crystals containing Gadolinium ions possess magnetic properties and can be good host materials for rare earth ions, but the growth of fluoride crystal nanofibers through electrospinning is not straight forward.

The electrospinning technique naturally leads to the growth of amorphous materials; therefore, a calcination step is always needed to obtain crystal nanofibers. Usually, the whole procedure to grow oxide fibers comprises three main steps:

(1) preparation of a suitable solution based on polymer and inorganic salt;
(2) electrospinning of this solution to obtain polymer precursor fibers;
(3) high temperature calcination of the precursor fibers to dissolve the polymer matrix and obtain crystallization of the material.

The calcination temperature must be optimized for every composition, but it usually is somewhat lower than what required in the solid-state synthesis of the same compound, and this represents another advantage of this technique. During this calcination step, the polymer precursor evaporates, thus leading to a shrinkage of the fibers whose diameter can be as low as 100 nm. This step, sometimes, also give the fibers a curly morphology and usually leads to the formation of crystalline grains connected and distributed along the fiber length.

**Figure 4:**
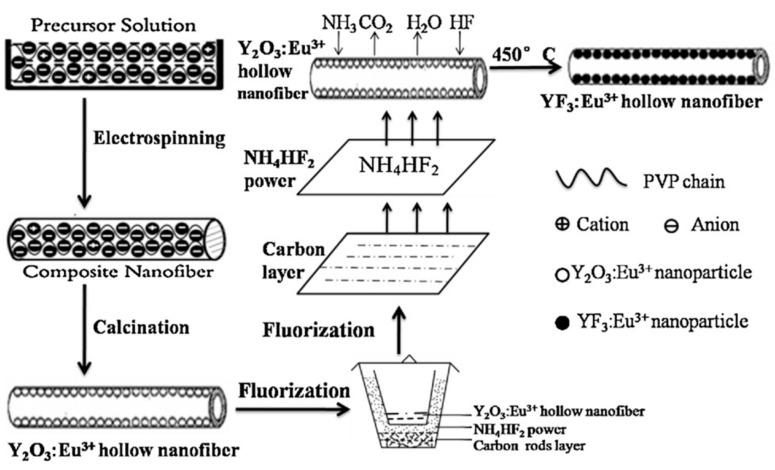
Schematic of the process to grow YF_3_:Eu^3+^ hollow nanofibers. Reprinted with permission from [29]. Copyright © 2012 Elsevier B.V.

The growth of fluoride fibers is usually accomplished through the growth of oxide fibers, first, with a further fluorination step that is usually carried out with the so-called double crucible method [29]. This method is basically a solid-gas reaction with the oxide fibers placed in the inner crucible and a fluorinating agent like NH_4_HF_2_ in the outer crucible together with carbon rods that play an important role in the reduction process. The physical separation of the oxide fibers and the fluorinating agent prevents the fibers from morphology damage. Fluoride fibers obtained with this method usually retain the original morphology of the oxide precursor fibers with no further shrinking of the diameter. The schematic of this process is depicted in Figure 4.

Grown fibers are usually characterized with different techniques to assess their crystal quality and morphology. These usually include X-ray diffraction (XRD), dispersive X-ray analysis (EDX), scanning electron microscopy (SEM) and transmission electron microscopy (TEM). In some cases, other techniques are employed like differential thermal analysis (DTA), thermogravimetric DTA (TG-DTA) and Fourier-Transform Infrared Spectroscopy (FTIR).

**Figure 5:**
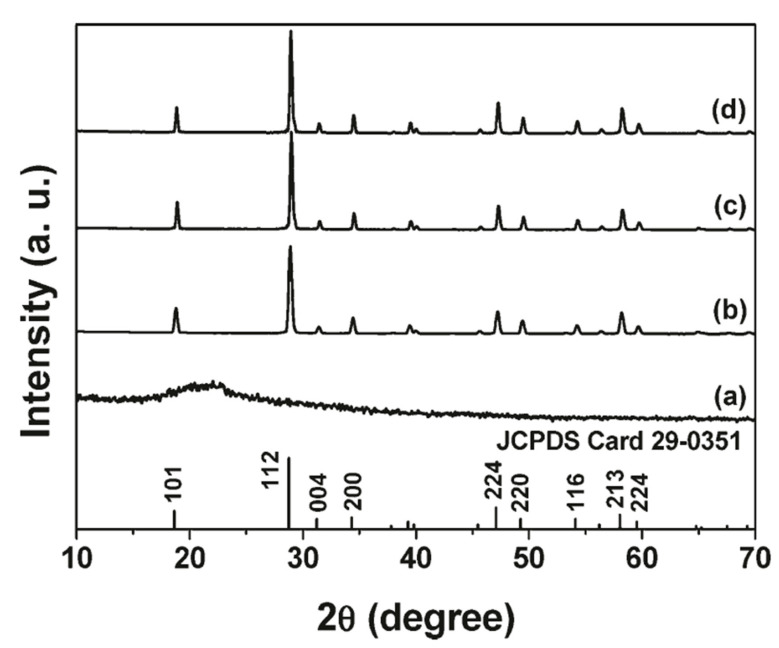
X-ray diffraction patterns for CaMoO_4_:5 mol% Ln^3+^ nanofibers: (**a**) as-formed precursor fibers; (**b**) the CaMoO_4_:5 mol% Tb^3+^ fibers annealed at 800 °C, (**c**) CaMoO_4_:5 mol%Eu^3+^ annealed at 800 °C, (**d**) CaMoO_4_:5 mol% Dy^3+^ annealed at 800 °C; and the JCPDS card 29-0351 of CaMoO_4_ for comparison. Reprinted with permission from [30]. Copyright © 2009 American Chemical Society.

Samples grown with this technique usually show a single crystalline phase, but fibers are usually made of crystalline grains distributed along the fiber length. An example of the good crystalline phase that can be obtained with this technique is reported in Figure 5. Diameters range from a few microns to even less than 100 nm depending on the host material and growth conditions. The morphology of the samples can show a smooth surface with randomly oriented straight fibers, but in some cases the surface is rough, and fibers become curly after the calcination process.

**Figure 6:**
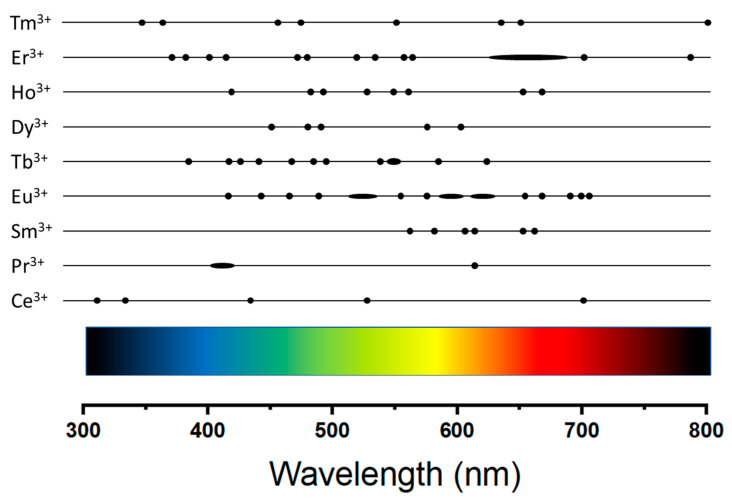
Approximate peak emission lines of the rare earth ions in the visible region.

Here, we review the main results obtained in the electrospinning growth of fluoride or oxide crystal matrixes doped with rare earth ions. Table 1 and Table 2 show the list of oxide and fluoride fibers grown by electrospinning, respectively. Tables report the crystal composition listed in alphabetical order and the dopant/s ions in the second column. The mean diameters of the obtained fibers are reported in the third column. In the case of fluoride fibers (Table 2) column 3 shows if the fibers are composed of single crystalline phase or are made of a polymer matrix with fluoride crystalline nanoparticles embedded. Figure 6 shows the approximate spectral position of the main emission lines of rare earth ions in the visible region.

## 4. Electrospun Rare Earth Doped Crystal Fibers

### 4.1. Electrospun Fibers with Stokes Emissions

Stokes emission leads to an emission wavelength longer than the excitation wavelength. The energy mismatch is called quantum defect and represents energy loss inside the material, unless a bilinear process is involved; therefore, in general it should be kept low. In fact, this is used to convert the radiation within a certain range, usually from UV to VIS or from blue to red emission mainly for lightning applications. This is the case for white LED emission that is usually obtained by coating a InGaN blueLED with a suitable phosphor to convert part of the blue emission into red radiation to obtain an overall emission within the white light region. Other possible applications range from scintillation to projection field emission display (FED), cathode ray tubes (CRTs), photocatalitic activity, electroluminescence (EL), persistent luminescence, antibacterial activity, biolabeling, etc.

Useful materials should have a possibly broad and intense absorption in the UV or blue region and a strong emission in the rest of the visible region. Many rare earth ions are suitable for this purpose either alone or as co-dopant with other rare earth or transition metal ions that have a broad absorption band and can efficiently transfer this energy to the emitting ion. In this case, the host material is required to be chemically stable and withstand high irradiation intensity without showing solarization effects even over long times. Oxide crystals are the preferred choice in most cases, but fluoride crystals can compete in these requirements, even if their growth is generally more complicated. Many rare earth ions show luminescent features that are suitable for the above-mentioned applications and most of them have been studied in nanofiber form of different compositions because the high surface-to-volume ratio of these shape opens new applicative possibilities that have been only partially explored up to now [32,34,78]. The main results published in this field are reported schematically in Table 1 and Table 2 for oxide and fluoride crystals, respectively. In this section, I will review these results with the focus on the emitting ion and its emission behaviour.

Ce is the first rare earth ion of the series; therefore, it only has one electron in the 4f shell and this gives rise to only one 4f multiplet that lies in the mid-infrared. This ion also possesses absorption bands in the UV and visible regions that are due to electronic excitation to the 4d shell. These transitions are not parity-forbidden; therefore, they have much shorter lifetimes and correspondingly larger cross sections and are usually also spectrally much broader than intra-4f transitions. In fact, Ce^3+^ ions are usually exploited for their very large absorption and emission features in the UV and visible regions but they also have a strong interaction with the crystalline lattice and this leads to large energy losses inside the material. Moreover, the exact location of these bands is strongly host-dependent and their properties are well-studied both in bulk and nanocrystalline hosts [115]. In nano fiber morphology, Ce^3+^ optical properties have been studied either as single dopant in TiO_2_ [67], YAG [77,78] and ZnO [21] or in conjunction with Tb^3+^ in LaPO_4_ [52], YAG [74] and Y_2_SiO_5_ [85]. It has also been incorporated in other crystal hosts without optical characterization because, in these cases, the focus was on the characterization of other functional property of the material, for example the magnetic properties of SrRe_0.6_Fe_11.4_O_19_ [59], the photocatalitic activity of TiO_2_ [64], the antibacterial activity of Ce_2_O_3_/TiO_2_ [70] or CeO_2_ [40].

Ce^3+^ absorption band that usually lies in the UV-VIS region is about 100 nm broad and its location is strongly host-dependent, for example it is peaked at 455 nm in YAG, at around 350 nm in TiO_2_, at 278 nm in LaPO_4_, 248 nm in Y_2_SiO_5_ and in the 300–400 nm region in ZnO. The 455 nm broad absorption of the Ce:YAG phosphor perfectly overlaps the blue emission of commercial LEDs and can be exploited for converting these devices into white-LED (WLED). Moreover, the nanofiber or nanobelt morphology is well suited to be used as coating material. Bright visible emission has been obtained from the Ce^3+^ ions in YAG with a very broad emission band peaked at 520–530 nm [74,77]. In this case, optimization of the concentration indicates 1% as the best choice for maximizing the intensity of the emission [77]. Moreover, this material has already been tested in a realistic device made of a commercial blue LED coated with the nanofibers for converting part of the blue radiation from a 450 nm LED to obtain white light emission. After optimization the authors demonstrated a luminous efficiency of 62 lm/W with various correlated color temperatures (CCT) in the 7281 to 5266 K range as a function of the phosphor layer thickness [78]. Other Ce^3+^ doped host crystals have been tested for other applications like LaPO_4_ that shows cathodoluminescence emission [52].

The strong absorption band of Ce^3+^ is also interesting as a sensitizer. For example, the Ce^3+^—Tb^3+^, codoping has been characterized in YAG [74], LaPO_4_ [52], Tb_2_(WO_4_) [60] and Y_2_SiO_5_ [85] nanofibers with interesting color-tuning properties and long emission lifetimes. In fact, Tb^3+^ ions possess a series of narrow emission lines in the green-red region typical of intra 4f shell transitions and can be used also as a single dopant in oxide nanofibers; Absorption of Tb^3+^ ions usually consists of two main features: a parity-allowed band in the 200–300 nm region, corresponding to the 4f^8^→ 4f^7^5d^1^ transition and some narrower 4f→ 4f bands in the 300–400 nm region that can be ascribed to the absorption from the ^7^F_6_ ground state to various excited states, while the emission consists of the transitions from the ^5^D_3,4_ to the ^7^F_J_ (J = 3, 4, 5, 6) multiplets. For these reasons, the Ce^3+^—Tb^3+^ system has been studied also in fluoride nanofibers. In particular, Ce^3+^, Tb^3+^: NaYF_4_ nanoparticles have been embedded in PVP nanofibers with average diameter 400 nm [108]. Under 254 nm excitation, visible emission has been obtained at various emission peaks from violet to red. This demonstrates an efficient energy transfer from Ce^3+^ to Tb^3+^ ions. In the same paper, Ag-nanoparticles have also been added to study possible plasmonic interaction to enhance the fluorescence features of Tb^3+^ ions. Unfortunately, no surface plasmon resonance (SPR) effects have been observed.

These interesting spectroscopic properties have triggered the study of the emission features of Tb^3+^ ions alone in many different host matrixes in nanofiber form: CaMoO_4_ [30], Ca_4_Y_6_(SiO_4_)_6_O [37], CaWO_4_ [39], Ga_2_O_3_ [43], Gd_2_O_3_ [44] LaBO_3_ [48], LaOBr [49], LaOCl [51], LaPO_4_ [52], Tb_2_(WO_4_)_3_ [60], YAG [73], Y_2_O_3_ [83], Y_2_SiO_5_ [85], ZnAl_2_O_4_ [91], ZrO_2_ [96] and YF_3_ [22]. In all these cases the diameter of the fibers obtained does not exceed 300 nm with a few exceptional cases of average diameter even less than 100 nm [44,49]. Excitation is usually performed to the 4f^8^→ 4f^7^5d^1^ band, rarely, in the 4f→ 4f bands [22,43]. Emission lines can cover the whole visible region from violet (382 nm) to red (621 nm), with color-tunable possibilities [22,30,49,73,91] and lifetimes range from tens of microseconds up to even tens of milliseconds [22,30,37,39,49,52,60,73,83]. In some cases, the cathodoluminescence properties of these compounds has been tested with interesting results [30,37,39,51,52,60,91]. Given the number of energy levels of the Tb^3+^ ion, detrimental bilinear energy transfer processes can be triggered by relatively high doping levels. In fact, the optimum doping concentration has been determined to be in the 3at. % to 7at. % range [22,43,44,49,51,96].

**Figure 7:**
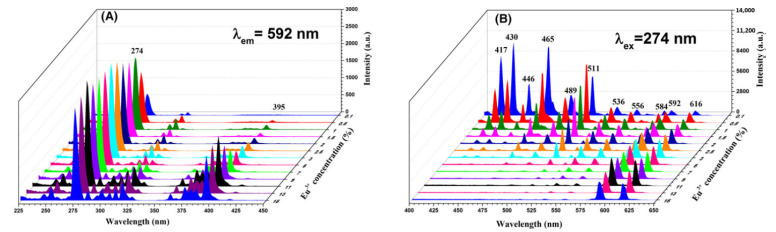
Excitation (**A**) and emission (**B**) spectra of NaGdF_4_:x%Eu^3+^ (x = 0.1, 0.5, 1, 1.5, 2, 2.5, 3, 5, 7, 9, 11, 13, 15) nanofibers. Reprinted with permission from [13]. Copyright © 2017 John Wiley and Sons.

Tb^3+^ ions can also be used as a sensitized for Eu^3+^ ions, or as emitter in conjunction with Eu^3+^ [60]. In fact, Eu^3+^ ions, also possess a series of visible emission lines that originate from the ^5^D_0_ and ^5^D_1_ multiplets with good color-tuning properties [30,72,82], that make them very interesting for lightening applications. In fact, Eu-doped nanofiber mats have already been tested in realistic LED devices [32,34] and Eu^3+^ is probably the mostly studied rare earth ion in nanofiber matrixes [1,13,19,29,30,32,34,35,37,41,42,45,47,48,51,53,54,55,61,62,67,68,76,79,80,81,82,84,86,87,88,89,91,101,112] including some nanobelt structures [72] and hollow structures [82]. In most of these cases, diameter of the nanofibers was less than 300 nm, with a few exceptions [35,42,76]. In some cases, exceptionally small diameter nanofibers have been reported, such as 50–100 nm in Y (V, P) O_4_ [89], 60–70 nm in TiO_2_ [62], 86.5 ± 0.5 nm in GdF_3_ [19], 20–100 nm in TiO_2_ [68], 30–50 nm in YVO_4_ [86], 40 nm for YBO_3_ nanowires [80] and even 30 nm in YVO_4_ [87]. Luminescence is usually studied after UV excitation of the 5d Eu^3+^ absorption band that lies in between 250 and 350 nm in most compounds, but 394 nm in YF_3_ [29,112] and 395 in YAG [79]. Emission of Eu^3+^ ions is composed of several host-dependent peaks with wavelengths in the 500–720 nm region which locate its emission in the yellow-red region [30,34,47,48,51,72,82]. In some fluoride matrixes at high Eu^3+^ doping level, Eu^3+^ fluorescence shows some emission lines in the blue region, as well. This is the case for NaGdF_4_ [13] and GdF_3_ [19] with interesting color-tuning capabilities. An example of the rich emission spectrum of Eu^3+^ and its color tuning potentiality as a function of the doping level is reported in Figure 7.

When concentration color-tuning is not possible, the addiction of a second doping ion has been tested. In fact, the addition of Tm^3+-^, Sm^3+-^ or Tb^3+-^ ions shifts the emission color from blue, to red, to green [51]. Since most of the emission lines originate from ^5^D_0_ reported lifetimes usually refer to the decay of this level and concentration-dependent values range from 0.5 ms to more than 5 ms in oxide hosts [30,35,37,45,47,53,55,80,81,82,84,86,87,89] and from 7 to 12 ms in fluorides [13,19,29,112]. In very few cases the lifetime of the ^5^D_j_ levels with j > 0 has been reported. For example in YVO_4_ the lifetime of ^5^D_1_ is reported to be around 7.5 μs [89], in NaGdF_4_ lifetimes of ^5^D_3_, ^5^D_2_, ^5^D_1_ are 2.38, 4.04, 6.36 ms, respectively and in GdF_3_ lifetimes of ^5^D_3_, ^5^D_2_, ^5^D_1_ are 2.26, 3.58, 3.88 ms, respectively. Concentration optimization of the luminescence intensity yelds an optimum concentration of Eu^3+^ ions usually in the range of 5–8% [47,53,54,72,89] before the onset of concentration quenching effects, but in some compounds such as Y_2_O_3_, Y_2_O_2_S and Lu_2_O_2_S lower values (around 3%) have been reported [55,82] and in Gd_2_O_3_ no concentration quenching effects have been observed up to 10% Eu^3+^ concentration. Moreover, in some cases polarized emission have been observed and characterized [76,81] and in Ca (Sr)Al_2_Si_2_O_8_ persistent luminescence from Eu^3+^ ions has been observed with a lifetime of 157.12 s. Some of these compounds possess bifunctional properties. This is the case, for example of Gd-containing fluoride compositions that have been tested for their optical-magnetical properties [13,19] and YAG-Al_2_O_3_ reinforced fibers whose luminescence features have been characterized under tensile stress. In this case a linear red shift of the luminescence peaks has been observed at increasing tensile stresses up to 300 MPa.

Another interesting rare earth ion with Stokes emission is Dy^3+^ which has been studied in nanofibers of different compositions such as CaMoO_4_, GdVO_4_, YAG, Y_2_O_3_, Y (V, P) O_4_, Ca_2_RE_8_(SiO_4_)_6_O_2_ (RE = Y, Gd) [30,38,47,75,83,89] and in conjunction with Eu^3+^ codoping in NaGdF_4_ [99] with emission lines located in the blue-yellow region. In all these cases, diameters of the nanofibers are in the 50–250 nm range except for YAG nanobelts that are 3850 ± 900 nm large [75]. Excitation of Dy^3+^ is usually accomplished in the charge-transfer band in the UV region and emissions are composed by a series of sharp lines originating from the ^4^F_9/2_ level. The exact location depends on the host crystal and they are mainly concentrated in the 450–600 nm region. Depending on the host crystal the emission is located either in the yellow–green region [30,47] or in the geen-blue region [38,75] with excellent color tuning capabilities [30,99]. The lifetime of the emitting level strongly depends on both the host crystal and the doping concentration and varies between 70 and 1500 μs. An interesting advantage of Dy^3+^ is that it can be doped at a relatively high level (5–7%) without evident concentration quenching effects. This is the case of CaMoO_4_ [30], YAG [75] and Y (V, P) O_4_ [89], while in GdVO_4_ concentrations higher than 2% already show a decrease of the luminescence intensity [47]. In some cases, this dopant has also been tested for cathodoluminescence applications [30,38,47,89].

Sm^3+^ and Pr^3+^ are other dopants that shows various Stokes emission bands concentrated in long-wavelength part of the visible region. The emission of Sm^3+^ has already been characterized in GdVO_4_, LaOCl, TiO_2_, Y_2_O_3_ and Y (V, P) O_4_ [47,51,63,83,89]. ZnO has been grown mainly for its photocatalitic activity [94]. Diameters of the grown fibers range from very small ones (less than 200 nm) [47,83,89] to relatively large ones (more than 500 nm) [63,94]. Excitation of Sm ions usually happens in the UV region and emission lines range from 567 to 660 nm [47,63,89] which locate its emission in the yellow-red region with optimum concentration in the 2–3% range [47,63] and lifetime of about 0.5 msec [47,89]. Another interesting feature is the polarized emission recorded from TiO_2_: Sm nanofibers [63].

The emission of Pr^3+^ doped nanofibers has been observed and characterized in CaTiO and TiO_2_. Diameters were 500 and 150 nm, respectively, and UV excitation leads to emission at around 400 nm in TiO_2_ and around 600 nm in CaTiO_3_. In this last case, the emission shows an afterglow with time constant of about 38 sec.

Er^3+^ is one of the few rare earth ions that can be used either as a Stokes or anti-Stokes emitter. The Stokes emission is obtained after UV or 488 nm excitation and emission lines are usually located in the green part of the visible spectrum, but it also has a strong emission line in the near infrared at around 1500 nm that can be interesting for telecom applications and has been observed in GeO_2_ and SnO_2_ electrospun fibers [46,56]. Visible lines have been observed and characterized in GeO_2_, SnO_2_, TiO_2_, ZnO, YAG, NaGdF_4_ [17,46,50,62,65,66,67,69,94,100]. Diameter of the grown fibers range from very small (less than 200 nm) [62,65,66,67,69] to medium (in between 200 and 600 nm) [17,46,56,94]. In some cases, fibers have been grown to assess the photocatalitic properties and no luminescence characterization has been carried out [65,69,94]. It may be worth mentioning the multifunctional properties of NaGdF_4_ that has been tested as a dual-drug carrier platform [100]. Since the growth of fluoride crystal nanofibers is more complicated, a common approach with fluorides is the growth of polymer nanofibers with fluoride nanoparticles embedded inside. As a result, multifunctional nanofibers resulted to be good drug-releasing agents for in-vivo orthotopic chemotherapy and also served as upconversion fluorescence/magnetic resonance dual-model imaging materials.

Other less studied rare earth ions that show interesting Stokes emission are Ho^3+^ and Nd^3+^. Ho^3+^ ions have been introduced in a mixed-anion crystal, HoOF. This composition can be excited in the UV region at around 290 nm [42] to obtain several emission lines from 416 nm to 660 nm. The emission of Nd^3+^ ions is located in the near infrared, instead, with three main emission bands at around 900 nm, 1000 nm and 1300 nm [92]. In this case, this infrared emission in nanosized shape is interesting for biological applications. To this purpose, the low cytotoxicity of Nd: ZnAl_2_O_4_ for human cells has assessed the suitability of this composition as a biological marker. Nd-doped nanofibers can also be interesting for their photocatalitic activity. In fact, it has been demonstrated that the addiction of Nd enhances the photocatalitc activity of TiO_2_ nanofibers [64].

Stokes emission can also be obtained from divalent rare earth ions or transition metal ions. This is the case, for example of Eu^2+^ [114] which can even show persistent afterglow emission in Ca (Sr)Al_2_Si_2_O_8_ [35] and SrAl_2_O_4_ [36]. In these cases, diameter of the grown fibers was 193 ± 1 nm in the case of BaFCl [114] and around 500–600 nm for the other two compounds. UV excitation lead to UV/visible emission at 387, 428 nm and 515 nm, respectively. In BaFCl the intensity of the emission shows a maximum at an 8% doping level and the lifetime of the emitting level varied from 0.5 to 2 ms depending on the concentration [114]. Both the other two compounds showed a persistent afterglow with decay time as long as 157 sec and 202 sec, respectively.

Eu^2+^ ions have also been studied as sensitizers for other rare earth ions like Nd^3+^ in Ba_5_Si_8_O_21_ [31], Dy^3+^ in Ca_2_MgSi_2_O_7_ [33], Ca (Sr)Al_2_Si_2_O_8_ [35], SrAl_2_O_4_ [36], Sr_2_MgSi_2_O_7_ [58], CaAl_2_Si_2_O_8_ [36] and SrAl_2_O_4_ [57]. In all these cases, long persistent visible luminescence has been observed with an afterglow lifetime which, in the best case, exceeded 42 min [31]. Eu:BaFCl nanofibers emit an interesting band at around 387 nm with a maximum intensity at 8% Eu^2+^ concentration that corresponds to a lifetime of 2.52 μs.

Transition metal ions usually show shorter lifetimes with respect to rare earth ions and do not undergo bilinear processes, therefore, they can be efficient Stokes emitting centers and have already been doped into electrospinned fibers. This is the case, for example of Mn^4+^: CaAl_12_O_19_ [32], Ni^2+^: ZnAl_2_O_4_ [90], Cr^3+^: ZnAl_2_O_4_ [91], Mn^2+^: ZnGa_2_O_4_ [93] and Cu^2+^: ZnS [95]. In some cases, even prototypal characterization in realistic devices has been performed. For example, the combination of Ce:YAG and Mn^4+^:CaAl_12_O_19_ nanofibers permitted to obtain bright warm light emission with a color rendering index (CRI) of 88.5, a CCT of 4553 K and Commission Internationale de I’Eclairage (CIE) color coordinates of (0.360, 0.334) [32].

### 4.2. Electrospun Fibers with Anti-Stokes Emissions

**Figure 8:**
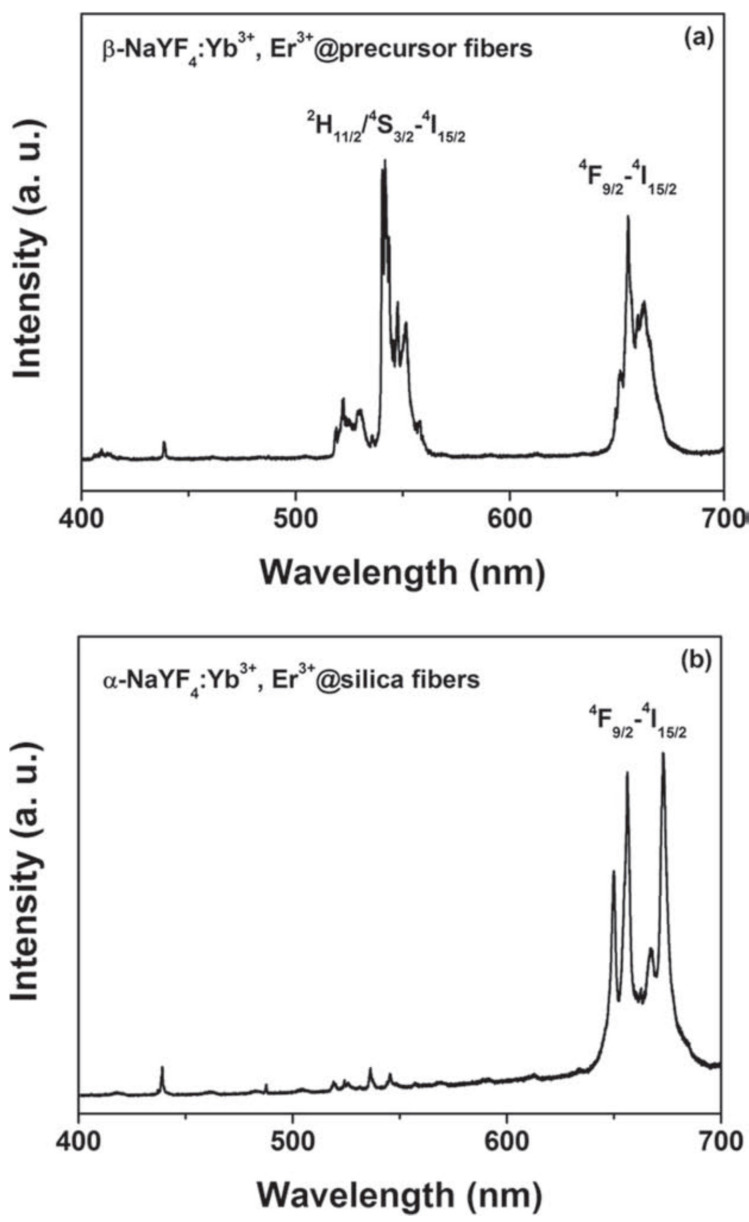
Up-conversion emission spectra of (**a**) β -NaYF_4_:Yb^3+^, Er^3+^@precursor fibers and (**b**) α -NaYF_4_:Yb^3+^, Er^3+^@silica fibers excited by 980 nm NIR laser. [102]. Copyright © 2011 WILEY-VCH Verlag GmbH & Co. KGaA, Weinheim.

Anti-Stokes emission indicates the emission of photons with a wavelength shorter than the excitation one. This is possible with rare earth ions through bilinear processes like upconversion. In such a process, an excited ion gives all or part of its energy to a nearby ions that is already in an excited state. The result is one ion excited to an energy level higher than both the starting ones. Since this interaction needs two excited ions in nearby locations, its probability increases both with the pump intensity and the lifetime of the levels involved. It is well known that rare earth ions in fluoride matrixes usually show longer lifetimes than in oxide matrixes and this is the main reason why bilinear processes are much more efficient in fluoride crystals. Another advantage of using fluoride materials is their lower phonon energy which decreases non radiative rates which can be detrimental both to the efficiency of the bilinear process and to the intensity of the emission. In fact, the great majority of anti-Stokes emission in electrospun fibers has been demonstrated in fluoride materials. An example of an efficient anti-Stokes emission is given in Figure 8.

Among the various rare earth ions that can show anti-Stokes emission, Er^3+^ is one of the favourite because it possesses a large number of energy levels that gives rise to many energy matching that can trigger upconversion processes. In fact, under 980 nm excitation Er^3+^ ions usually show bright visible luminescence with the main peaks in the green and red region and sometimes, in the blue [116]. This emission can be enhanced by codoping the material with Yb^3+^ ions because Yb^3+^ ions possess only one energy level that strongly absorbs the radiation at around 980 nm and efficiently transfers it to Er^3+^ ions without introducing detrimental effects. The visible luminescence of Er^3+^ ions is particularly intense in fluoride matrixes and this explains the interest in the growth of fluoride crystal electrospinned nanofibers doped with Er^3+^ ions like BaY_2_F_8_ [97], BaYF_5_ [16], Ba_4_Y_3_F_17_ [98] or in embedding Er^3+^: NaYF_4_ fluoride nanoparticles into polymer fibers [12,60,103,104,105,106,107,109]. In some cases, other ions or non-fluoride crystals have been investigated, like Er^3+^: LaOBr nanobelts [50], Yb^3+^, Tm^3+^: YF_3_ [111] and Yb^3+^, Er^3+^: YAG [71]. Most of the grown fibers have diameters smaller than or around 300 nm except for a few cases [104,106,107] and for nanobelts [71,109]. Some of these materials also possess multifunctional properties that have been investigated, like magnetic properties when added with Gd or Fe [12,109] or drug delivery properties [12,60,103,104]. As for the emission properties, when investigated, the quadratic dependence of the emission intensity versus the incident power demonstrates that the population of the emitting level happens through a bilinear process and the upconversion efficiency are largely preserved in the fibers with respect to the bare nanoparticles [105]. Moreover, in most of the compounds this emission shows good color tunable capabilities [71,97,103,104,107] that can be interesting for imaging and labelling applications. Since Er^3+^ possess many energy levels, concentration-quenching effects prevent the use of high doping levels. The optimum doping level is usually found at around 5% or lower [21,41].

Very recently, Yb has been used as sensitizer for other rare earth ions that can undergo upconversion processes and emit in visible radiation. This is the case of Yb, Tm:La_2_Ti_2_O_7_ [110] that shows excellent upconversion properties and Yb,Ho:Y_2_T_i2_O_7_ [113] whose potentialities for temperature sensing have been assessed.

It is worth mentioning that works on fibers with anti-Stokes emission are much more advanced from an applicative point of view with respect to the Stokes-emitting fibers which mainly focus on the growth procedure, morphological, structural and spectroscopic characterization of the grown materials. Anti-Stokes emission is quite interesting for biological imaging applications because it permits to use an excitation wavelength in the transmission window of tissues and gives rise to visible emission where sensitive detection apparatuses are the state-of-the-art. Moreover, cellular autofluorescence is a Stokes process, therefore detecting the anti-Stokes emission permits to completely eliminate the cellular autofluorescence background. To this aim the cytotoxicity and cell uptake behavior are crucial parameters. These has been evaluated to be good for example in Yb^3+^, Er^3+^: NaYF_4_@silica fibers that have also shown an UC luminescence intensity that increases with the released amount of drug [60,104] and this can permit a real-time monitoring of the drug release process. Moreover, the same group in 2013 has shown that these fibers can deliver two different types of drugs with distinct releasing properties. These results indicate these materials as promising multi-functional drug carriers for drug delivery and cell imaging applications.

## 5. Applications

Most of the works presented here, mainly deal with the spectroscopic investigation of the emitting rare earth ions, some present different types of bifunctionality, but very few of them are really application-oriented. The main applications that have really been tested are in the biomedical field. Among these, the drug-delivery capability of electrospinned fibers is probably the most promising. For example, this has been observed and characterized in [12,100,102,103,104] with good results; in fact, the drug delivery process can be monitored through the observation of the fluorescence of the material. This opens very interesting applicative scenarios, provided that the materials are proven not to be cytotoxic as found by [92]. Moreover, electrospun nanofibers have been also tested for their anti-bacterial activity [70]. In this case, the spectral properties of rare earth ions do not play any active role, but the presence of Ce^3+^ is used to enhance the anti-bacterial activity which can be used for the disinfection of food pathogens.

Another promising applicative field is the use of the nanofibers as photocatalysts [64,69,94]. Waste water from texile industry can contain pigments or dyes that can cause severe pollution problems. Nowadays, the quest for an efficient and sustainable technology to solve this problem is still open and photocatalysis has attracted much attention in the last few decades. To this aim, rare-earth-doped nanofibers are very attractive because it has been proven that rare-earth ions enhance the overall photodegradation capability of nanofibrous composites.

Moreover, some rare earth ions possess interesting magnetic properties and the possibility to combine these magnetic properties with the optical emission of materials in nanoshaped form is very intriguing. This has been explored, for example, in [12,13,19,59,99,100,109], with interesting results in terms of magnetization, magnetization hysteresis or paramagnetic performance and results show that these magnetic properties can be tuned by changing the doping concentration, sometimes also in conjunction with a change of the colour of the emission. These findings can be useful in different fields such as for solid-state lasers, lighting, displays and magnetic resonance imaging.

Rare earth doped crystals are also good as scintillators, for this reason, some groups investigated the cathodo- or radio- luminescence performance of the nanocrystalline fibers [17,54,55,76] in view of a possible use, for example, as porous scintillators for the detection of ionizing radiation of flowing fluids.

Last but not least, many of the papers presented in this review are focused on the coloured emission of the nanofibers for possible applications in the lightning field and, most of all, as wavelength converters for blue LEDs to obtain white light emission. Interesting color tuning characteristic can be obtained with many compounds by changing the dopant level with a great potential for applications in this field and one paper even presents a realistic test of nanofibrous materials as wavelength converters to obtain a white LED with a luminous efficiency as high as 62 lm/W and correlated colour temperatures varying from 7281 K to 5266 K by changing the thickness of the phosphor layer [78].

## 6. Conclusions

In the last two decades, electrospinning growth of rare-earth-doped crystal nanofibers has brought about excellent advancements: many different materials among the oxide and fluoride classes have been successfully grown and doped with a variety of rare earth ions. From these compounds, Stokes and anti-Stokes emissions have been obtained with good results in terms of color tuning capabilities, emission efficiency and lifetime values. In a few cases, multifunctional capabilities and application potentialities have been tested with very good results.

Therefore, after two decades of electrospinning growth of rare earth doped nanofibers, we can say that research has demonstrated that this technology is mature for a step forward towards taking this technology out of the lab in the above-mentioned fields.

## Figures and Tables

**Table 1:** Oxides grown with the electrospinning technique: physical and optical properties.

Crystal	Active Ion	Diameter (nm)	l_p_ (nm)	l_em_ (nm)	Ref.
Ba_5_Si_8_O_21_	Eu^2+^, Nd^3+^	2000	341	365–650	[31]
CaAl_12_O_19_	Mn^4+^	500	325, 390, 457	600–700	[32]
Ca_2_MgSi_2_O_7_	Eu^2+^, Dy^3+^	2500	378	430–650	[33]
CaMoO_4_	Eu^3+^	80–150	280	591, 615	[30]
CaMoO_4_	Tb^3+^	80–150	283	543	[30]
CaMoO_4_	Dy^3+^	80–150	285	478, 487, 576	[30]
CaSi_2_O_2_N_2_	Eu^3+^	200–300	-	500-600	[34]
Ca (Sr)Al_2_Si_2_O_8_	Eu^3+^	500	263, 393	580, 529, 614, 654	[35]
Ca (Sr)Al_2_Si_2_O_8_	Eu^2+^	500	330	428	[35]
Ca (Sr)Al_2_Si_2_O_8_	Eu^2+^, Dy^3+^	500	330	428	[35]
CaTiO_3_	Pr^3+^	500	330	615	[36]
CaAl_2_Si_2_O_8_	Eu^2+^, Dy^3+^	200–800	350	428	[36]
Ca_4_Y_6_(SiO_4_)_6_O	Eu^3+^	120–260	277	540,580, 588, 616, 655, 705	[37]
Ca_4_Y_6_(SiO_4_)_6_O	Tb^3+^	120–260	235	418, 440, 488, 545, 585, 624	[37]
Ca_2_RE_8_(SiO_4_)_6_O_2_ (RE = Y, Gd)	Pb^2+^, Dy^3+^	140–190	254	347, 482, 574	[38]
CaWO_4_	Tb^3+^	50–150	249	382, 415, 436, 466, 545, 585, 619	[39]
CeO_2_	Ce^3+^	300	-	-	[40]
CuAlO_2_	Eu^3+^	-	365, 465	587, 610, 654, 690	[41]
EuOF	Eu^3+^	800	290	579, 591, 609, 654, 706	[42]
Ga_2_O_3_	Eu^3+^	180–300	325	598, 620, 665, 704	[1]
Ga_2_O_3_	Tb^3+^	100–300	325	491, 550, 591, 625	[43]
Gd_2_O_3_	Tb^3+^	80	274	493, 545, 595, 619	[44]
Gd_2_O_3_	Eu^3+^	60–150	254	587, 615, 629	[45]
GeO_2_	Er^3+^	388 ± 186	488	1540	[46]
GdVO_4_	Eu^3+^	100–160	276	620	[47]
GdVO_4_	Dy^3+^	100–160	276	484, 574	[47]
GdVO_4_	Sm^3+^	100–160	276	567, 604, 649	[47]
HoOF	Ho^3+^	750	290	416, 488, 528, 555; 652, 660	[42]
LaBO_3_	Eu^3+^	100	254	550–700	[48]
LaBO_3_	Tb^3+^	100	254	485, 543, 582, 622,	[48]
LaOBr	Tb^3+^	90 ± 15	253	418, 438, 486, 543	[49]
LaOBr	Er^3+^	3060 ± 420	980	522, 541, 667,1500 (@ex.532)	[50]
LaOCl	Eu^3+^	100–200	295	531, 554, 577, 594, 615, 648, 698	[51]
LaOCl	Sm^3+^	100–200	-	-	[51]
LaOCl	Tb^3+^	100–200	234	382, 415, 438, 486, 543, 584, 621	[51]
LaOCl	Tb^3+^, Eu^3+^	100–200	488	543, 615	[51]
LaOCl	Tm^3+^, Eu^3+^	100–200	488	382, 415, 438, 486, 543, 584, 621	[51]
LaPO_4_	Ce^3+^	75–150	278	318, 336,	[52]
LaPO_4_	Tb^3+^	75–150	216	489, 543, 585, 620	[52]
LaPO_4_	Ce^3+^, Tb^3+^	75–150	278	487, 543, 583, 619	[52]
La_2_W_2_O_9_	Eu^3+^	184 ± 19	288	533, 570-700	[53]
Lu_2_O_3_	Eu^3+^	90–180	237	612	[54]
Lu_2_O_2_S	Eu^3+^	252	345	610	[55]
SnO_2_	Er^3+^	~590	488	1540	[56]
SrAl_2_O_4_	Eu^2+^, Dy^3+^	180–200	346–375	509 broadband	[57]
SrAl_2_O_4_	Eu^2+^	600	348	515	[36]
SrAl_2_O_4_	Eu^2+^, Dy^3+^	600	348	515	[36]
Sr_2_MgSi_2_O_7_	Eu^2+^, Dy^3+^	1500	360	410–590	[58]
SrRe_0.6_Fe_11.4_O_19_	Ce^3+^	200–300	-	-	[59]
Tb_2_(WO_4_)_3_	Tb^3+^	80–150	280	488, 543, 585, 619	[60]
Tb_2_(WO_4_)_3_	Tb^3+^, Eu^3+^	80–150	280	488, 543, 592, 652, 702	[60]
TiO_2_	Eu^3+^	80–100	395	580, 595, 615	[61]
TiO_2_	Eu^3+^	60–70	CL	580, 595, 615, 652, 700	[62]
TiO_2_	Er^3+^	60–80	CL	567, 528, 669	[62]
TiO_2_	Sm^3+^	1000	330	580, 610, 660	[63]
TiO_2_	Ce^3+^	700	-	-	[64]
TiO_2_	Nd^3+^	340	-	-	[64]
TiO_2_	Er^3+^	160	-	-	[65]
TiO_2_	Er^3+^	75	325	528, 567, 669, 815	[66]
TiO_2_	Eu^3+^	150	UV	~650-750	[67]
TiO_2_	Er^3+^	150	UV	~700	[67]
TiO_2_	Ce^3+^	150	UV	~700	[67]
TiO_2_	Pr^3+^	150	UV	~400–420	[67]
TiO_2_	Eu^3+^	20–100	325	600, 621, 667, 695	[68]
TiO_2_/SiO_2_	Er^3+^	-	-	-	[69]
Ce_2_O_3_/TiO_2_	Ce^3+^	300	-	-	[70]
YAG	Yb^3+^, Er^3+^	1800 ± 370	980	522, 554, 648	[71]
YAG	Eu^3+^	3250	235	592, 597, 611, 632	[72]
YAG	Tb^3+^	166 ± 20	274	486, 544, 587, 623	[73]
YAG	Ce^3+^, Tb^3+^	4090 ± 410	273	490, 544, 584	[74]
YAG	Dy^3+^	3850 ± 900	352	452, 484, 583	[75]
YAG	Er^3+^	590 ± 190	381	510-580, 630-690	[17]
YAG	Eu^3+^	400-500	238	592, 612, 650, 708	[76]
YAG	Ce^3+^	300	470	530	[77]
YAG	Ce^3+^	200–250	450	500–600	[78]
YAG-Al_2_O_3_	Eu^3+^	300	395	570–720	[79]
YBO_3_	Eu^3+^	40	245	591, 609, 624	[80]
Y_2_O_3_	Eu^3+^	300	260	578, 592, 612, 652, 688	[81]
Y_2_O_3_	Eu^3+^	184 ± 26	260	581, 588, 594, 612, 632	[82]
Y_2_O_3_	Tb^3+^	35–260	304	~500	[83]
Y_2_O_3_	Sm^3+^	35–260	-	-	[83]
Y_2_O_3_	Dy^3+^	35–260	458	~600	[83]
Y_2_O_3_	Eu^3+^	200–400	241	609	[84]
Y_2_O_2_S	Eu^3^	137 ± 18	260	515, 540, 557, 584, 588, 596, 618, 628	[82]
Y_2_SiO_5_	Ce^3+^	70–140	367	437	[85]
Y_2_SiO_5_	Tb^3+^	70–140	248	489, 542, 585, 625	[85]
Y_2_SiO_5_	Ce^3+^, Tb^3+^	70–140	367	437, 489, 542, 585, 625	[85]
YVO_4_	Eu^3+^	30–50	280	~600	[86]
YVO_4_	Eu^3+^	30	280	~600	[87]
PEO/YVO_4_	Eu^3+^	300	290	593, 615, 650, 698	[88]
Y (V, P) O_4_	Eu^3+^	50–100	290	538, 587, 618, 698	[89]
Y (V, P) O_4_	Sm^3+^	50–100	280	567, 602, 649	[89]
Y (V, P) O_4_	Dy^3+^	50–100	280	483, 573	[89]
ZnAl_2_O_4_	Ni^2+^	120	576	1000–1400	[90]
ZnAl_2_O_4_	Cr^3+^	140–230	400	689	[91]
ZnAl_2_O_4_	Eu^3+^	140–230	278	570–720	[91]
ZnAl_2_O_4_	Tb^3+^	140–230	227	380, 415, 438, 490, 545, 587	[91]
ZnAl_2_O_4_	Nd^3+^	~200	808	905, 1064, 1330	[92]
ZnGa_2_O_4_	Mn^2+^	96	246	505	[93]
ZnO	Ce^3+^	350	325, 350, 365	400–600	[21]
ZnO	Er^3+^	600	-	-	[94]
ZnO	Sm^3+^	600	-	-	[94]
ZnS	Cu^2+^	300	315	450–600	[95]
ZrO_2_	Tb^3+^	250	325	488, 543, 584, 620	[96]

**Table 2:** Fluorides grown with the electrospinning technique: physical and optical properties.

Crystal	Dopant/s	Diameter (nm)	l_p_(nm)	l_em_(nm)	Ref
BaY_2_F_8_	Er^3+^	160 ± 16	980	523, 540, 652	[97]
BaYF_5_	Er^3+^	110 ± 11	980	522, 540, 651	[16]
Ba_4_Y_3_F_17_	Er^3+^	84 ± 5	980	523, 541, 652	[98]
NaY/GdF_4_	Yb^3+^, Er^3+^	75–200	980	510–570, 630–700	[12]
NaGdF_4_	Eu^3+^	231 ± 4	274	417, 430, 446, 465, 489, 511, 536, 556, 584, 592, 616	[13]
NaGdF_4_	Dy^3+^, Eu^3+^	246 ± 52	274	478, 570, 592, 616	[99]
NaGdF_4_	Yb^3+^, Er^3+^	-	274	521, 542, 652	[100]
NaGdF_4_	Eu^3+^	200	273	525-650	[101]
NaYF_4_	Yb^3+^, Er^3+^	About 200	980	562, 655, 663, 673	[102]
NaYF_4_	Yb^3+^, Er^3+^	About 300	980	510–575, 660–675	[103]
NaYF_4_	Yb^3+^, Er^3+^	300–750	980	522, 542, 655	[104]
NaYF_4_	Yb^3+^, Er^3+^	316 ± 66	980	523, 539, 656	[105]
NaYF_4_	Yb^3+^, Er^3+^, Tm^3+^	300–800	980	476, 451, 550, 649, 660-740	[106]
NaYF_4_	Yb^3+^, Er^3+^	400	980	375, 405, 538,520, 655	[107]
NaYF_4_	Tb^3+^, Ce^3+^	400	254	280-420, 375, 414, 438, 465, 490, 544, 586, 622	[108]
NaYF_4_	Yb^3+^, Er^3+^	260	980	479, 487, 542, 789	[109]
La_2_Ti_2_O_7_	Tm^3+^, Yb^3+^	500–1000	977	550, 650	[110]
YF_3_	Yb^3+^, Tm^3+^	200–300	980	291, 346, 362, 453, 477, 642, 802	[111]
YF_3_	Tb^3+^	148 ± 23	367	490, 543, 588, 620	[22]
YF_3_	Eu^3+^	211 ± 29	394	587, 593, 615, 620	[112]
YF_3_	Eu^3+^	197 ± 34	394	555, 587, 593, 615, 620, 651, 692	[29]
Y_2_Ti_2_O_7_	Ho^3+^, Yb^3^	300–400	977	550, 650	[113]
GdF_3_	Eu^3+^	86.5 ± 0.5	274	418, 430, 445, 464, 489, 510, 538, 555, 587, 594, 615	[19]
BaFCl	Eu^2+^	193 ± 1	275	387	[114]

## Data Availability

Data sharing not applicable.

## References

[1] Zhao J., Zhang W., Xie E., Ma Z., Zhao A., Liu Z. (2011). Structure and photoluminescence of β-Ga_2_O_3_:Eu^3+^ nanofibers prepared by electrospinning. Appl. Surf. Sci..

[2] Pisignano D. (2013). Polymer Nanofibers Building Blocks for Nanotechnology.

[3] Persano L., Camposeo A., Pisignano D. (2015). Active polymer nanofibers for photonics, electronics, energy generation and micromechanics. Prog. Polym. Sci..

[4] Angammana C.J., Jayaram S.H. (2016). Fundamentals of electrospinning and processing technologies. Part. Sci. Technol..

[5] Mirjalili M., Zohoori S. (2016). Review for application of electrospinning and electrospun nanofibers technology in textile industry. J. Nanostruct. Chem..

[6] Wu H., Pan W., Lin D., Li H. (2012). Electrospinning of ceramic nanofibers: Fabrication, assembly and applications. J. Adv. Ceram..

[7] Huang Z.-M., Zhang Y.-Z., Kotaki M., Ramakrishna S. (2003). A review on polymer nanofibers by electrospinning and their applications in nanocomposites. Compos. Sci. Technol..

[8] Denker B., Shklovsky E. (2013). Handbook of Solid-State Lasers: Materials, Systems and Applications.

[9] Kaminskiĭ A.A. (1996). Crystalline Lasers: Physical Processes and Operating Schemes.

[10] Cornacchia F., Toncelli A., Tonelli M. (2009). 2-μm lasers with fluoride crystals: Research and development. Prog. Quantum Electron..

[11] Wen S., Zhou J., Zheng K., Bednarkiewicz A., Liu X., Jin D. (2018). Advances in highly doped upconversion nanoparticles. Nat. Commun..

[12] Liu M., Liu H., Sun S., Li X., Zhou Y., Hou Z., Lin J. (2014). Multifunctional Hydroxyapatite/Na(Y/Gd)F_4_:Yb^3+^, Er^3+^ Composite Fibers for Drug Delivery and Dual Modal Imaging. Langmuir.

[13] Li D., Ma Q., Song Y., Xi X., Dong X., Yu W., Wang J., Liu G. (2017). A novel strategy to achieve NaGdF_4_:Eu^3+^ nanofibers with color-tailorable luminescence and paramagnetic performance. J. Am. Ceram. Soc..

[14] Liu Y.-J., Zhang H.-D., Yan X., Zhao A.-J., Zhang Z.-G., Si W.-Y., Gong M.-G., Zhang J.-C., Long Y.-Z. (2016). Effect of Ce doping on the optoelectronic and sensing properties of electrospun ZnO nanofibers. RSC Adv..

[15] Wilhelm S. (2017). Perspectives for Upconverting Nanoparticles. ACS Nano.

[16] Liu Y., Li D., Ma Q., Dong X., Xi X., Yu W., Wang X., Wang J., Liu G. (2016). Er^3+^ doped BaYF_5_ nanofibers: Facile construction technique, structure and upconversion luminescence. J. Mater. Sci. Mater. Electron..

[17] Chen Z., Trofimov A.A., Jacobsohn L.G., Xiao H., Kornev K.G., Xu D., Peng F. (2017). Permeation and optical properties of YAG:Er^3+^ fiber membrane scintillators prepared by novel sol–gel/electrospinning method. J. Sol. Gel. Sci. Technol..

[18] Szewczyk P.K., Gradys A., Kim S.K., Persano L., Marzec M., Kryshtal A., Busolo T., Toncelli A., Pisignano D., Bernasik A. (2020). Enhanced Piezoelectricity of Electrospun Polyvinylidene Fluoride Fibers for Energy Harvesting. ACS Appl. Mater. Interfaces.

[19] Li D., Ma Q., Song Y., Xi X., Dong X., Yu W., Wang J., Liu G. (2016). Tunable multicolor luminescence and white light emission realized in Eu^3+^ mono-activated GdF_3_ nanofibers with paramagnetic performance. RSC Adv..

[20] Xi X., Ma Q., Dong X., Wang J., Yu W., Liu G. (2015). Flexible Janus Nanofiber to Help Achieve Simultaneous Enhanced Magnetism-Upconversion Luminescence Bifunction. IEEE Trans. Nanotechnol..

[21] Ma Q., Wang J., Dong X., Yu W., Liu G. (2015). Magnetic-upconversion luminescent bifunctional flexible coaxial nanoribbon and Janus nanoribbon: One-pot electrospinning preparation, structure and enhanced upconversion luminescent characteristics. Chem. Eng. J..

[22] Li D., Dong X., Yu W., Wang J., Liu G. (2013). Fabrication and luminescence of YF_3_:Tb^3+^ hollow nanofibers. J. Mater. Sci. Mater. Electron..

[23] Sun B., Long Y.Z., Zhang H.D., Li M.M., Duvail J.L., Jiang X.Y., Yin H.L. (2014). Advances in three-dimensional nanofibrous macrostructures via electrospinning. Prog. Polym. Sci..

[24] Kaerkitcha N., Chuangchote S., Sagawa T. (2016). Control of physical properties of carbon nanofibers obtained from coaxial electrospinning of PMMA and PAN with adjustable inner/outer nozzle-ends. Nanoscale Res. Lett..

[25] Ma Q., Yu W., Dong X., Yang M., Wang J., Liu G. (2015). Flexible Tricolor Flag-liked Microribbons Array with Enhanced Conductive Anisotropy and Multifunctionality. Sci. Rep..

[26] Burger C., Hsiao B.S., Chu B. (2006). Nanofibrous Materials and Their Applications. Annu. Rev. Mater. Res..

[27] Loscertales I.G. (2002). Micro/Nano Encapsulation via Electrified Coaxial Liquid Jets. Science.

[28] Díaz J.E., Barrero A., Márquez M., Loscertales I.G. (2006). Controlled Encapsulation of Hydrophobic Liquids in Hydrophilic Polymer Nanofibers by Co-electrospinning. Adv. Funct. Mater..

[29] Li D., Yu W., Dong X., Wang J., Liu G. (2013). Synthesis and luminescence properties of YF_3_:Eu^3+^ hollow nanofibers via the combination of electrospinning with fluorination technique. J. Fluor. Chem..

[30] Hou Z., Chai R., Zhang M., Zhang C., Chong P., Xu Z., Li G., Lin J. (2009). Fabrication and Luminescence Properties of One-Dimensional CaMoO_4_:Ln^3+^ (Ln = Eu, Tb, Dy) Nanofibers via Electrospinning Process. Langmuir.

[31] Yao Y., Zhou Z., Ye F. (2017). Properties of a novel Ba_5_Si_8_O_21_:Eu^2+^,Nd^3+^ phosphor: Bulk and 1D nanostructure with PVP synthesized by sol-gel and electrospinning. J. Alloy. Compd..

[32] Liu Z., Yuwen M., Liu J., Yu C., Xuan T., Li H. (2017). Electrospinning, optical properties and white LED applications of one-dimensional CaAl_12_O_19_:Mn^4+^ nanofiber phosphors. Ceram. Int..

[33] Ye F., Dong S., Tian Z., Yao S., Zhou Z., Wang S. (2015). Fabrication and characterization of long-persistent luminescence/polymer (Ca_2_MgSi_2_O_7_:Eu^2+^, Dy^3+^/PLA) composite fibers by electrospinning. Opt. Mater..

[34] Gu Y., Zhang Q., Wang H., Li Y. (2011). CaSi_2_O_2_N_2_:Eu nanofiber mat based on electrospinning: Facile synthesis, uniform arrangement, and application in white LEDs. J. Mater. Chem..

[35] Dong G., Xiao X., Liu X., Qian B., Ma Z., Chen D., Qiu J. (2009). Preparation and Optical Properties of Long Afterglow Europium-Doped Ca(Sr)Al_2_Si_2_O_8_ Electrospun Nanofibers. J. Electrochem. Soc..

[36] Dong G., Xiao X., Zhang L., Ma Z., Bao X., Peng M., Zhang Q., Qiu J. (2011). Preparation and optical properties of red, green and blue afterglow electrospun nanofibers. J. Mater. Chem..

[37] Peng C., Li G., Kang X., Li C., Lin J. (2011). The fabrication of one-dimensional Ca_4_Y_6_(SiO_4_)_6_O:Ln^3+^ (Ln=Eu, Tb) phosphors by electrospinning method and their luminescence properties. J. Colloid Interface Sci..

[38] Peng C., Kang X., Li G., Hou Z., Li C., Lin J. (2011). Fabrication and Luminescence Properties of Ca_2_RE_8_(SiO_4_)_6_O_2_:Pb^2+^,Dy^3+^ (RE = Y, Gd) One-Dimensional Phosphors by Electrospinning Method. J. Electrochem. Soc..

[39] Hou Z., Li C., Yang J., Lian H., Yang P., Chai R., Cheng Z., Lin J. (2009). One-dimensional CaWO_4_ and CaWO_4_:Tb^3+^ nanowires and nanotubes: Electrospinning preparation and luminescent properties. J. Mater. Chem..

[40] Cui Q., Dong X., Wang J., Li M. (2008). Direct fabrication of cerium oxide hollow nanofibers by electrospinning. J. Rare Earths.

[41] Liu Y., Gong Y., Mellott N.P., Wang B., Ye H., Wu Y. (2016). Luminescence of Delafossite-Type CuAlO_2_ Fibers with Eu Substitution for Al Cations. Sci. Technol. Adv. Mater..

[42] Wang H.Y., Yang Y., Wang Y., Zhao Y.Y., Li X., Wang C. (2009). Luminescent Properties of Rare-Earth Oxyfluoride Nanofibers Prepared via Electrospinning. J. Nanosci. Nanotech..

[43] Zhao J., Zhang W., Xie E., Liu Z., Feng J., Liu Z. (2011). Photoluminescence properties of β-Ga_2_O_3_:Tb^3+^ nanofibers prepared by electrospinning. Mater. Sci. Eng. B.

[44] Du P., Song L., Xiong J., Xi Z., Jin D., Wang L. (2011). Preparation and the luminescent properties of Tb^3+^ -doped Gd_2_O_3_ fluorescent nanofibers via electrospinning. Nanotechnology.

[45] Yu H., Li Y., Song Y., Wu Y., Chen B., Li P. (2016). Preparation and luminescent properties of Gd_2_O_3_:Eu^3+^ nanofibres made by electrospinning. Ceram. Int..

[46] Wu J., Coffer J.L. (2007). Emissive Erbium-Doped Silicon and Germanium Oxide Nanofibers Derived from an Electrospinning Process. Chem. Mater..

[47] Li X., Yu M., Hou Z., Li G., Ma P., Wang W., Cheng Z., Lin J. (2011). One-dimensional GdVO_4_:Ln^3+^ (Ln=Eu, Dy, Sm) nanofibers: Electrospinning preparation and luminescence properties. J. Solid State Chem..

[48] Qin C., Qin L., Chen G., Lin T. (2013). One-dimensional Eu^3+^ and Tb^3+^ doped LaBO_3_ nanofibers: Fabrication and improved luminescence performances. Mater. Lett..

[49] Ma W., Dong X., Wang J., Yu W., Liu G. (2014). Study on terbium doped lanthanum oxybromide luminescent nanoribbons and nanofibers. J. Mater. Sci. Mater. Electron..

[50] Ma W., Dong X., Wang J., Yu W., Liu G. (2014). Preparation of LaOBr:Er^3+^ Up-conversion Luminescent Nanobelts by Electrospinning Then Bromination. J. Elec. Mater..

[51] Li G., Hou Z., Peng C., Wang W., Cheng Z., Li C., Lian H., Lin J. (2010). Electrospinning Derived One-Dimensional LaOCl:Ln^3+^ (Ln = Eu/Sm, Tb, Tm) Nanofibers, Nanotubes and Microbelts with Multicolor-Tunable Emission Properties. Adv. Funct. Mater..

[52] Hou Z., Wang L., Lian H., Chai R., Zhang C., Cheng Z., Lin J. (2009). Preparation and luminescence properties of Ce^3+^ and/or Tb^3+^ doped LaPO_4_ nanofibers and microbelts by electrospinning. J. Solid State Chem..

[53] Song K., Li G.-M. (2016). Electrospinning synthesis, characterization and luminescence properties of La_2_W_2_O_9_:Eu^3+^ nanofibers. J. Mater. Sci. Mater. Electron..

[54] Li X., Yu M., Hou Z., Wang W., Li G., Cheng Z., Chai R., Lin J. (2010). Preparation and luminescence properties of Lu_2_O_3_:Eu^3+^ nanofibers by sol–gel/electrospinning process. J. Colloid Interface Sci..

[55] Zhang B., Zou H., Song Y., Guan H., Zhou X., Shi Z., Sheng Y. (2017). Electrospinning fabrication and luminescence properties of Lu_2_O_2_S:Eu^3+^ fibers. Cryst. Eng. Comm..

[56] Wu J., Coffer J.L. (2007). Strongly Emissive Erbium-Doped Tin Oxide Nanofibers Derived from Sol Gel/Electrospinning Methods. J. Phys. Chem. C.

[57] Cheng Y., Zhao Y., Zhang Y., Cao X. (2010). Preparation of SrAl_2_O_4_:Eu^2+^,Dy^3+^ fibers by electrospinning combined with sol–gel process. J. Colloid Interface Sci..

[58] Ye F., Dong S., Tian Z., Yao S., Zhou Z., Wang S. (2013). Fabrication of the PLA/Sr_2_MgSi_2_O_7_:Eu^2+^,Dy^3+^ long-persistent luminescence composite fibers by electrospinning. Opt. Mater..

[59] Li C.-J., Wang J.-N. (2010). Electrospun SrRe_0.6_Fe_11.4_O_19_ magnetic nanofibers: Fabrication and characterization. Mater. Lett..

[60] Hou Z., Cheng Z., Li G., Wang W., Peng C., Li C., Ma P., Yang D., Kang X., Lin J. (2011). Electrospinning-derived Tb_2_(WO_4_)_3_:Eu^3+^ nanowires: Energy transfer and tunable luminescence properties. Nanoscale.

[61] Bianco A., Cacciotti I., Fragalá M.E., Lamastra F.R., Speghini A., Piccinelli F., Malandrino G., Gusmano G. (2010). Eu-Doped Titania Nanofibers: Processing, Thermal Behaviour and Luminescent Properties. J. Nanosci. Nanotechnol..

[62] Cacciotti I., Bianco A., Pezzotti G., Gusmano G. (2011). Synthesis, thermal behaviour and luminescence properties of rare earth-doped titania nanofibers. Chem. Eng. J..

[63] Dong G., Xiao X., Chi Y., Qian B., Liu X., Ma Z., Ye S., Wu E., Zeng H., Chen D. (2009). Polarized Luminescence Properties of TiO_2_:Sm^3+^ Microfibers and Microbelts Prepared by Electrospinning. J. Phys. Chem. C.

[64] Hassan M.S., Amna T., Yang O.-B., Kim H.-C., Khil M.-S. (2012). TiO_2_ nanofibers doped with rare earth elements and their photocatalytic activity. Ceram. Int..

[65] Lee D.Y., Kim B.-Y., Cho N.-I., Oh Y.-J. (2011). Electrospun Er^3+^–TiO_2_ nanofibrous films as visible light induced photocatalysts. Curr. Appl. Phys..

[66] Jia C.W., Zhao J.G., Duan H.G., Xie E.Q. (2007). Visible photoluminescence from Er^3+^-doped TiO_2_ nanofibres by electrospinning. Mater. Lett..

[67] Wang H., Wang Y., Yang Y., Li X., Wang C. (2009). Photoluminescence properties of the rare-earth ions in the TiO_2_ host nanofibers prepared via electrospinning. Mater. Res. Bull..

[68] Zhao J., Jia C., Duan H., Sun Z., Wang X., Xie E. (2008). Structural and Photoluminescence Properties of Europium-Doped Titania Nanofibers Prepared by Electrospinning Method. J. Alloy. Compd..

[69] Wang J., An X., Yu Y., Li X., Ge M. (2018). Er-Doped Titanium Dioxide/Silicon Dioxide Fibres with Enhanced Photodegradation Performance. Micro Nano Lett..

[70] Hassan M.S., Amna T., Al-Deyab S.S., Kim H.-C., Oh T.-H., Khil M.-S. (2012). Toxicity of Ce_2_O_3_/TiO_2_ composite nanofibers against S. aureus and S. typhimurium: A novel electrospun material for disinfection of food pathogens. Colloids Surf. A Physicochem. Eng. Asp..

[71] Bi F., Dong X., Wang J., Liu G. (2014). Facile Electrospinning Preparation and Up-Conversion Luminescence Performance of Y_3_Al_5_O_12_:Er^3+^,Yb^3+^ Nanobelts. J. Inorg. Organomet. Polym..

[72] Bi F., Dong X., Wang J., Liu G. (2015). Electrospinning Preparation and Photoluminescence Properties of Y_3_Al_5_O_12_:Eu^3+^ Nanobelts. Mat. Res..

[73] Bi F., Dong X., Wang J., Liu G. (2015). Electrospinning preparation and photoluminescence properties of Y_3_Al_5_O_12_:Tb^3+^ nanostructures: Electrospinning preparation of Y_3_Al_5_O_12_:Tb^3+^ nanostructures. Luminescence.

[74] Bi F., Gai G., Dong X., Xiao S., Wang J., Liu G., Zhao L., Wang L. (2017). Electrospinning preparation and photoluminescence properties of Y_3_Al_5_O_12_:Ce^3+^, Tb^3+^ nanobelts. J. Mater. Sci. Mater. Electron..

[75] Bi F., Gai G., Dong X., Xiao S., Liu G., Zhao L., Wang L. (2017). Facile electrospinning preparation and luminescence performance of color adjustable Y_3_Al_5_O_12_:Dy^3+^ nanobelts. J. Mater. Sci. Mater. Electron..

[76] Dong G., Xiao X., Chi Y., Qian B., Liu X., Ma Z., Wu E., Zeng H., Chen D., Qiu J. (2010). Size-dependent polarized photoluminescence from Y_3_Al_5_O_12_:Eu^3+^ single crystalline nanofiber prepared by electrospinning. J. Mater. Chem..

[77] Suryamas A.B., Munir M.M., Iskandar F., Okuyama K. (2009). Photoluminescent and crystalline properties of Y_3−x_Al_5_O_12_:Ce_x_^3+^ phosphor nanofibers prepared by electrospinning. J. Appl. Phys..

[78] Xu J., Zeng R., Gong Y. (2016). Preparation of electrospun YAG:Ce nanofiber-based phosphor layer for white LEDs application. Ceram. Int..

[79] He L., Pan L., Li W., Dong Q., Sun W. (2020). Spectral Response Characteristics of Eu^3+^ Doped YAG-Al_2_O_3_ Composite Nanofibers Reinforced Aluminum Matrix Composites. Opt. Mater..

[80] Song H., Yu H., Pan G., Bai X., Dong B., Zhang X.T., Hark S.K. (2008). Electrospinning Preparation, Structure, and Photoluminescence Properties of YBO_3_:Eu^3+^ Nanotubes and Nanowires. Chem. Mater..

[81] Dong G., Chi Y., Xiao X., Liu X., Qian B., Ma Z., Wu E., Zeng H., Chen D., Qiu J. (2009). Fabrication and optical properties of Y_2_O_3_:Eu^3+^ nanofibers prepared by electrospinning. Opt. Express.

[82] Han L., Pan M., Lv Y., Gu Y., Wang X., Li D., Kong Q., Dong X. (2015). Fabrication of Y_2_O_2_S:Eu^3+^ hollow nanofibers by sulfurization of Y_2_O_3_:Eu^3+^ hollow nanofibers. J. Mater. Sci. Mater. Electron..

[83] Li X., Chen Y., Qian Q., Liu X., Xiao L., Chen Q. (2012). Preparation and Photoluminescence Characteristics of Tb-, Sm- and Dy-Doped Y_2_O_3_ Nanofibers by Electrospinning. J. Lumin..

[84] Yu H., Song H., Pan G., Li S., Liu Z., Bai X., Wang T., Lu S., Zhao H. (2007). Preparation and Luminescent Properties of Europium-Doped Yttria Fibers by Electrospinning. J. Lumin..

[85] Wang L., Hou Z., Quan Z., Li C., Yang J., Lian H., Yang P., Lin J. (2009). One-Dimensional Ce^3+^- and/or Tb^3+^ -Doped X_1_-Y_2_SiO_5_ Nanofibers and Microbelts: Electrospinning Preparation and Luminescent Properties. Inorg. Chem..

[86] Yu H., Song H., Pan G., Qin R., Fan L., Zhang H., Bai X., Li S., Zhao H., Lu S. (2008). Preparation and Luminescent Properties of YVO_4_:Eu^3+^ Nanofibers by Electrospinning. J. Nanosci. Nanotechnol..

[87] Yu H., Song Y., Li Y., Wu Y., Chen B., Li P., Sheng C. (2016). Preparation and luminescent properties of one-dimensional YVO_4_: Eu nanocrystals. J. Mater. Sci. Mater. Electron..

[88] Chigome S., Abiona A.A., Ajao J.A., Kana J.B.K., Guerbous L., Torto N., Maaza M. (2010). Synthesis and Characterization of Electrospun Poly(ethylene oxide)/Europium-Doped Yttrium Orthovanadate (PEO/YVO_4_:Eu^3+^) Hybrid Nanofibers. Int. J. Polym. Mater..

[89] Hou Z., Yang P., Li C., Wang L., Lian H., Quan Z., Lin J. (2008). Preparation and Luminescence Properties of YVO_4_:Ln and Y(V,P)O_4_:Ln (Ln = Eu^3+^, Sm^3+^, Dy^3+^) Nanofibers and Microbelts by Sol−Gel/Electrospinning Process. Chem. Mater..

[90] Dong G., Liang M., Qin H., Chai G., Zhang X., Ma Z., Peng M., Qiu J. (2012). Controllable fabrication and broadband near-infrared luminescence of various Ni^2+^-activated ZnAl_2_O_4_ nanostructures by a single-nozzle electrospinning technique. Phys. Chem. Chem. Phys..

[91] Peng C., Li G., Geng D., Shang M., Hou Z., Lin J. (2012). Fabrication and luminescence properties of one-dimensional ZnAl_2_O_4_ and ZnAl_2_O_4_:A^3+^ (A=Cr, Eu, Tb) microfibers by electrospinning method. Mater. Res. Bull..

[92] Yang D., Zhao G., Pan Q., Liang M., Ma Z., Dong G., Chen D., Qiu J. (2013). Electrospun Nd^3+^-doped spinel nanoparticles/nanofibers with both excitation and emission wavelengths in the optical window of cells and tissues. Mat. Express.

[93] Wang L., Hou Z., Quan Z., Lian H., Yang P., Lin J. (2009). Preparation and luminescence properties of Mn^2+^-doped ZnGa_2_O_4_ nanofibers via electrospinning process. Mater. Res. Bull..

[94] Pascariu P., Cojocaru C., Olaru N., Samoila P., Airinei A., Ignat M., Sacarescu L., Timpu D. (2019). Novel rare earth (RE-La, Er, Sm) metal doped ZnO photocatalysts for degradation of Congo-Red dye: Synthesis, characterization and kinetic studies. J. Environ. Manag..

[95] Wang H., Lu X., Zhao Y., Wang C. (2006). Preparation and characterization of ZnS:Cu/PVA composite nanofibers via electrospinning. Mater. Lett..

[96] Xie Y., Ma Z., Liu L., Su Y., Zhao H., Liu Y., Zhang Z., Duan H., Li J., Xie E. (2010). Oxygen defects-modulated green photoluminescence of Tb-doped ZrO_2_ nanofibers. Appl. Phys. Lett..

[97] Liu Y., Li D., Ma Q., Yu W., Xi X., Dong X., Wang J., Liu G. (2016). A new scheme to acquire BaY_2_F_8_:Er^3+^ nanofibers with upconversion luminescence. J. Mater. Sci. Mater. Electron..

[98] Liu Y., Li D., Ma Q., Yu W., Xi X., Dong X., Wang J., Liu G. (2016). Fabrication of Novel Ba_4_Y_3_F_17_:Er^3+^ Nanofibers with Upconversion Fluorescence via Combination of Electrospinning with Fluorination. J. Mater. Sci. Mater. Electron..

[99] Li D., Ma Q., Xi X., Dong X., Yu W., Wang J., Liu G. (2017). Dy^3+^ and Eu^3+^ Co-doped NaGdF_4_ nanofibers endowed with bifunctionality of tunable multicolor luminescence and paramagnetic properties. Chem. Eng. J..

[100] Chen Y., Liu S., Hou Z., Ma P., Yang D., Li C., Lin J. (2015). Multifunctional electrospinning composite fibers for orthotopic cancer treatment in vivo. Nano Res..

[101] Dali L., Guolei W., Biao D., Xue B., Yu W., Hongwei S., Lin X. (2010). Electrospinning preparation and properties of NaGdF_4_:Eu^3+^ nanowires. Solid State Sci..

[102] Hou Z., Li C., Ma P., Li G., Cheng Z., Peng C., Yang D., Yang P., Lin J. (2011). Electrospinning Preparation and Drug-Delivery Properties of an Up-Conversion Luminescent Porous NaYF_4_:Yb^3+^, Er^3+^@Silica Fiber Nanocomposite. Adv. Funct. Mater..

[103] Hou Z., Li C., Ma P., Cheng Z., Li X., Zhang X., Dai Y., Yang D., Lian H., Lin J. (2012). Up-Conversion Luminescent and Porous NaYF_4_:Yb^3+^, Er^3+^@SiO_2_ Nanocomposite Fibers for Anti-Cancer Drug Delivery and Cell Imaging. Adv. Funct. Mater..

[104] Hou Z., Li X., Li C., Dai Y., Ma P., Zhang X., Kang X., Cheng Z., Lin J. (2013). Electrospun Upconversion Composite Fibers as Dual Drugs Delivery System with Individual Release Properties. Langmuir.

[105] Bao Y., Luu Q.A.N., Zhao Y., Fong H., May P.S., Jiang C. (2012). Upconversion Polymeric Nanofibers Containing Lanthanide-Doped Nanoparticles via Electrospinning. Nanoscale.

[106] Dong B., Song H., Yu H., Zhang H., Qin R., Bai X., Pan G., Lu S., Wang F., Fan L. (2008). Upconversion Properties of Ln^3+^ Doped NaYF_4_ /Polymer Composite Fibers Prepared by Electrospinning. J. Phys. Chem. C.

[107] Dong G., Liu X., Xiao X., Qian B., Ruan J., Yang H., Ye S., Chen D., Qiu J. (2009). Upconversion Luminescence of Er^3+^–Yb^3+^ Codoped NaYF_4_–PVP Electrospun Nanofibers. IEEE Photon. Technol. Lett..

[108] Dong G., Liu X., Xiao X., Qian B., Ruan J., Ye S., Yang H., Chen D., Qiu J. (2009). Photoluminescence of Ag Nanoparticle Embedded Tb^3+^/Ce^3+^ Codoped NaYF_4_/PVP Nanofibers Prepared by Electrospinning. Nanotechnology.

[109] Ma Q., Wang J., Dong X., Yu W., Liu G. (2014). Electrospinning Fabrication and Characterization of Magnetic-Upconversion Fluorescent Bifunctional Core–Shell Nanofibers. J. Nanopart. Res..

[110] Zhou R., Lin P., Yue E.B.P., Lin H., Yuan J., Zhao X. (2020). Hybrid excitation mechanism of upconversion fluorescence in hollow La_2_Ti_2_O_7_:Tm^3+^/Yb^3+^ submicron Fibers. J. Mater. Sci..

[111] Yang R., Song W., Liu S., Qin W. (2012). Electrospinning Preparation and Upconversion Luminescence of Yttrium Fluoride Nanofibers. CrystEngComm.

[112] Li D., Wang J., Dong X., Yu W., Liu G. (2013). Fabrication and Luminescence Properties of YF_3_:Eu^3+^ Hollow Nanofibers via Coaxial Electrospinning Combined with Fluorination Technique. J. Mater. Sci..

[113] Hu E. (2020). Fluorescent Thermal Feedback in Ho^3+^/Yb^3+^ Doped Y_2_Ti_2_O_7_ Electrospun Nanofibers. J. Electrochem. Soc..

[114] Zheng C., Li D., Ma Q., Song Y., Dong X., Wang X., Yu W., Wang J., Liu G. (2017). Novel Synthetic Strategy towards BaFCl and BaFCl:Eu^2+^ Nanofibers with Photoluminescence Properties. Chem. Eng. J..

[115] Jacobsohn L.G., Toncelli A., Sprinkle K.B., Kucera C.J., Ballato J. (2012). Spectral Engineering of LaF_3_:Ce^3+^ Nanoparticles: The Role of Ce^3+^ in Surface Sites. J. Appl. Phys..

[116] Sani E., Toncelli A., Tonelli M. (2006). Spectroscopy of Ce-Codoped Er:BaY_2_F_8_ Single-Crystals. Opt. Mater..

